# (*RS*)-Tricarbon­yl(η^4^-1,3-diacet­oxy-5,5-dimethyl­cyclo­hexa-1,3-diene)iron(0)

**DOI:** 10.1107/S1600536811041298

**Published:** 2011-10-12

**Authors:** Steffen Romanski, Jörg-M. Neudörfl, Hans-Günther Schmalz

**Affiliations:** a Department für Chemie der Universität zu Köln, Greinstrasse 4, 50939 Köln, Germany

## Abstract

In the title compound, [Fe(C_12_H_16_O_4_)(CO)_3_], the diene moiety of the mol­ecule is virtually planar, with a C—C—C—C torsion angle of −1.4 (2)°. The six-membered ring exhibits a boat conformation, with torsion angles of 46.2 (2) and 46.5 (3)° for a double-bond and the two attached C*sp* ^3^ atoms. The Fe atom is coordinated to all four of the diene C atoms, with bond lengths between 2.041 (2) and 2.117 (2) Å. The Fe(CO)_3_ tripod adopts a conformation with one CO ligand eclipsing the C*sp* ^3^—C*sp* ^3^ single bond.

## Related literature

For a short overview of CO as a signaling mol­ecule and of CO-releasing mol­ecules (CO-*RMs*), see: Choi & Otterbein (2002 ▶); Johnson *et al.* (2003 ▶); Alberto & Motterlini (2007 ▶); Mann & Motterlini (2007 ▶). For a very recent review of the biological activity of carbon monoxide gas and CO-*RMs*, see: Motterlini & Otterbein (2010 ▶). For the first use of the title compound as a CO-*RM*, see: Romanski *et al.* (2011 ▶). For a known synthesis of this mol­ecule in racemic form, see: Boháč *et al.* (1996 ▶). For a description of the Cambridge Structural Database, see: Allen (2002 ▶).
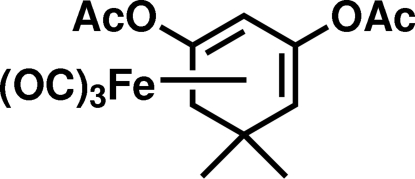


## Experimental

### 

#### Crystal data

[Fe(C_12_H_16_O_4_)(CO)_3_]
*M* *_r_* = 364.13
Monoclinic, 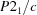

*a* = 10.9977 (6) Å
*b* = 11.9586 (5) Å
*c* = 13.0364 (5) Å
β = 108.739 (3)°
*V* = 1623.63 (13) Å^3^
*Z* = 4
Mo *K*α radiation
μ = 0.96 mm^−1^
*T* = 100 K
0.30 × 0.15 × 0.07 mm

#### Data collection

Nonius KappaCCD diffractometer
Absorption correction: multi-scan (*PLATON*; Spek, 2009 ▶) *T*_min_ = 0.700, *T*_max_ = 0.931
15588 measured reflections
3538 independent reflections
2814 reflections with *I* > 2σ(*I*)
*R*_int_ = 0.059

#### Refinement

*R*[*F* ^2^ > 2σ(*F* ^2^)] = 0.031
*wR*(*F* ^2^) = 0.078
*S* = 1.06
3538 reflections
212 parameters
H-atom parameters constrained
Δρ_max_ = 0.66 e Å^−3^
Δρ_min_ = −0.62 e Å^−3^

### 

Data collection: *COLLECT* (Hooft, 1998 ▶); cell refinement: *DENZO* (Otwinowski & Minor, 1997 ▶); data reduction: *DENZO*; program(s) used to solve structure: *SHELXS97* (Sheldrick, 2008 ▶); program(s) used to refine structure: *SHELXL97* (Sheldrick, 2008 ▶); molecular graphics: *SCHAKAL99* (Keller, 1999 ▶); software used to prepare material for publication: *PLATON* (Spek, 2009 ▶).

## Supplementary Material

Crystal structure: contains datablock(s) global, I. DOI: 10.1107/S1600536811041298/rk2295sup1.cif

Structure factors: contains datablock(s) I. DOI: 10.1107/S1600536811041298/rk2295Isup2.hkl

Additional supplementary materials:  crystallographic information; 3D view; checkCIF report

## Figures and Tables

**Fig. 1.**
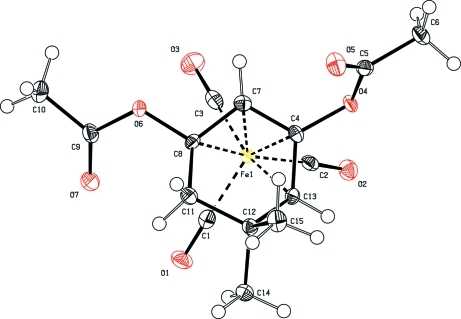
Molecular structure of title compound with the atom numbering scheme. Displacement ellipsoids are drawn at the 50% probability level. H atoms are presented as a small spheres of arbitrary radius.

**Fig. 2.**
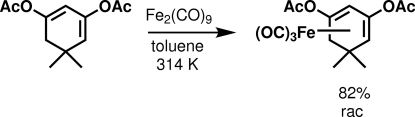
Synthesis path for the title compound.

## supplementary crystallographic information

### Comment

In recent years, CO has been recognized as an important signaling molecule (Choi & Otterbein, 2002). As CO is a toxic gas with a low bioavailability, an alternative way for its delivery is attractive and CO–releasing molecules (CO–*RMs*) proofed to be suitable tools (Johnson *et al.*, 2003; Alberto & Motterlini, 2007; Mann & Motterlini, 2007). CO–*RMs* as well as CO gas have successfully been used in various biological model systems to induce a broad scope of effects (Motterlini & Otterbein, 2010). In the context of our work we used acyloxy–diene iron carbonyl complexes as enzymatically–triggered CO–*RMs* (*ET*–CO*RMs*) representing a new class of CO–*RMs*. The biological evaluation of the title compound showed that it efficiently inhibits iNOS in murine macrophage cell line RAW264.7 and is to the best of our knowledge the most potent CO–*RMs* ever studied in this type of assay (Romanski *et al.*, 2011). Originally the complex was used as a precursor for non–racemic iron carbonyl complexes by exchange of one CO ligand with a chiral phosphinite and separation of the resulting diastereomers (Boháč *et al.*, 1996). The C—C and Fe—C bond length of the diene fit in with the already published dienylester ironcaronyl complex (Romanski *et al.*, 2011) and the data of the CSD database (Allen, 2002). In comparison to non–complexed dienes the inner bond of the diene system is noticeably shorter while the C═C are significantly longer. The contraction of the C—C single bond of the diene is most distinct in the case of the diacetoxy substituted title compound. Despite the electronic dissymmetry of the diene unit, the diene–Fe(CO)_3_ substructure is virtually symmetric (Fig. 1).

### Experimental

The title compound C_15_H_16_FeO_7_ was prepared in good yield by thermal complexation of 5,5–dimethylcyclohexa–1,3–diene–1,3–diyl diacetate with Fe_2_(CO)_9_ in toluene. In a dry, argon flushed 50 ml flask 1.34 mmol of 5,5–dimethylcyclohexa–1,3–diene–1,3–diyl diacetate and 4.13 mmol of Fe_2_(CO)_9_ were heated in 20 ml of dry toluene to 314 K for 4.5 h (Fig. 2). The solvent was evaporated and the crude mixture was purified by column chromatography (silica gel, ethylacetate/cyclohexane = 1:15) to give 1.10 mmol (82%) of the desired complex as a yellow oil that solidified after several weeks at 255 K. A portion of the complex was recrystallized by diffusion of a methanolic solution of the complex into water. Colourless crystals were obtained from the yellow oil.

### Refinement

All Hydrogen atoms were placed in geometrically idealized positions and refined with using riding model with C—H = 0.95Å and *U*_iso_(H) = 1.2*U*_eq_(C) for CH, C—H = 0.99Å and *U*_iso_(H) = 1.2*U*_eq_(C) for CH_2_, C—H = 0.98Å and *U*_iso_(H) = 1.5*U*_eq_(C) for CH_3_.

### Crystal data

**Table:**

[Fe(C_12_H_16_O_4_)(CO)_3_]	*F*(000) = 752
*M**_r_* = 364.13	*D*_x_ = 1.490 Mg m^−^^3^
Monoclinic, *P*2_1_/*c*	Mo *K*α radiation, λ = 0.71073 Å
Hall symbol: -P 2ybc	Cell parameters from 15588 reflections
*a* = 10.9977 (6) Å	θ = 2.0–27.0°
*b* = 11.9586 (5) Å	µ = 0.96 mm^−^^1^
*c* = 13.0364 (5) Å	*T* = 100 K
β = 108.739 (3)°	Prism, colourless
*V* = 1623.63 (13) Å^3^	0.3 × 0.15 × 0.07 mm
*Z* = 4	

### Data collection

**Table:**

Nonius KappaCCD diffractometer	3538 independent reflections
Radiation source: fine–focus sealed tube	2814 reflections with *I* > 2σ(*I*)
graphite	*R*_int_ = 0.059
φ and ω scans	θ_max_ = 27.0°, θ_min_ = 2.0°
Absorption correction: multi-scan (*PLATON*; Spek, 2009)	*h* = −14→13
*T*_min_ = 0.700, *T*_max_ = 0.931	*k* = −14→15
15588 measured reflections	*l* = −16→16

### Refinement

**Table:**

Refinement on *F*^2^	Primary atom site location: structure-invariant direct methods
Least-squares matrix: full	Secondary atom site location: difference Fourier map
*R*[*F*^2^ > 2σ(*F*^2^)] = 0.031	Hydrogen site location: inferred from neighbouring sites
*wR*(*F*^2^) = 0.078	H-atom parameters constrained
*S* = 1.06	*w* = 1/[σ^2^(*F*_o_^2^) + (0.0353*P*)^2^ + 0.1094*P*] where *P* = (*F*_o_^2^ + 2*F*_c_^2^)/3
3538 reflections	(Δ/σ)_max_ = 0.001
212 parameters	Δρ_max_ = 0.66 e Å^−^^3^
0 restraints	Δρ_min_ = −0.62 e Å^−^^3^

### Special details

**Table:**

Geometry. All s.u.'s (except the s.u. in the dihedral angle between two l.s. planes) are estimated using the full covariance matrix. The cell s.u.'s are taken into account individually in the estimation of s.u.'s in distances, angles and torsion angles; correlations between s.u.'s in cell parameters are only used when they are defined by crystal symmetry. An approximate (isotropic) treatment of cell s.u.'s is used for estimating s.u.'s involving l.s. planes.
Refinement. Refinement of *F*^2^ against ALL reflections. The weighted *R*–factor *wR* and goodness of fit *S* are based on *F*^2^, conventional *R*–factors *R* are based on *F*, with *F* set to zero for negative *F*^2^. The threshold expression of *F*^2^ > σ(*F*^2^) is used only for calculating *R*–factors(gt) *etc*. and is not relevant to the choice of reflections for refinement. *R*–factors based on *F*^2^ are statistically about twice as large as those based on *F*, and *R*–factors based on ALL data will be even larger.

### Fractional atomic coordinates and isotropic or equivalent isotropic displacement parameters (Å^2^)

**Table:**

	*x*	*y*	*z*	*U*_iso_*/*U*_eq_	
Fe1	0.66661 (3)	0.30437 (2)	0.424274 (19)	0.01185 (9)	
O1	0.94976 (13)	0.27963 (10)	0.43516 (10)	0.0147 (3)	
O2	0.86658 (15)	0.20345 (11)	0.26861 (10)	0.0227 (3)	
O3	0.68135 (13)	0.47430 (10)	0.60010 (9)	0.0141 (3)	
O4	0.86120 (14)	0.57754 (12)	0.67162 (11)	0.0229 (3)	
O5	0.41698 (15)	0.28056 (11)	0.46063 (11)	0.0204 (3)	
O6	0.75961 (16)	0.08729 (12)	0.52412 (12)	0.0301 (4)	
O7	0.57537 (14)	0.24054 (12)	0.19457 (10)	0.0220 (3)	
C1	0.84627 (19)	0.35591 (15)	0.42037 (14)	0.0126 (4)	
C2	0.8298 (2)	0.38305 (15)	0.52183 (14)	0.0142 (4)	
H2	0.8865	0.3589	0.5898	0.017*	
C3	0.72175 (19)	0.44864 (15)	0.51032 (14)	0.0127 (4)	
C4	0.64858 (19)	0.47985 (15)	0.40352 (13)	0.0123 (4)	
H4	0.5576	0.4736	0.3789	0.015*	
C5	0.72083 (19)	0.52401 (15)	0.32899 (14)	0.0141 (4)	
C6	0.8340 (2)	0.44474 (15)	0.33460 (14)	0.0152 (4)	
H6A	0.8193	0.4087	0.2633	0.018*	
H6B	0.9147	0.4883	0.3523	0.018*	
C7	0.6296 (2)	0.53454 (16)	0.21245 (14)	0.0180 (4)	
H7A	0.5586	0.5847	0.2110	0.027*	
H7B	0.5955	0.4606	0.1856	0.027*	
H7C	0.6765	0.5650	0.1663	0.027*	
C8	0.7709 (2)	0.64176 (16)	0.37013 (16)	0.0202 (5)	
H8A	0.6988	0.6890	0.3720	0.030*	
H8B	0.8126	0.6750	0.3213	0.030*	
H8C	0.8331	0.6360	0.4432	0.030*	
C9	0.9517 (2)	0.20967 (15)	0.35318 (15)	0.0174 (4)	
C10	1.0733 (2)	0.14411 (19)	0.38555 (17)	0.0262 (5)	
H10A	1.0677	0.0849	0.3323	0.039*	
H10B	1.0869	0.1106	0.4570	0.039*	
H10C	1.1453	0.1937	0.3887	0.039*	
C11	0.7642 (2)	0.53864 (15)	0.67896 (14)	0.0153 (4)	
C12	0.7140 (2)	0.55145 (17)	0.77265 (14)	0.0194 (5)	
H12A	0.6339	0.5944	0.7496	0.029*	
H12B	0.7777	0.5909	0.8318	0.029*	
H12C	0.6977	0.4774	0.7977	0.029*	
C13	0.5151 (2)	0.29061 (15)	0.44952 (14)	0.0148 (4)	
C14	0.7219 (2)	0.17132 (17)	0.48471 (15)	0.0186 (5)	
C15	0.6127 (2)	0.26595 (16)	0.28408 (15)	0.0157 (4)	

### Atomic displacement parameters (Å^2^)

**Table:**

	*U*^11^	*U*^22^	*U*^33^	*U*^12^	*U*^13^	*U*^23^
Fe1	0.01399 (17)	0.01153 (15)	0.01009 (14)	0.00017 (12)	0.00396 (11)	−0.00037 (10)
O1	0.0157 (8)	0.0155 (7)	0.0131 (6)	0.0044 (6)	0.0049 (6)	−0.0016 (5)
O2	0.0245 (9)	0.0251 (8)	0.0161 (7)	0.0045 (7)	0.0034 (6)	−0.0063 (6)
O3	0.0159 (8)	0.0169 (7)	0.0107 (6)	−0.0001 (6)	0.0061 (5)	−0.0034 (5)
O4	0.0199 (9)	0.0266 (8)	0.0235 (7)	−0.0063 (7)	0.0088 (6)	−0.0079 (6)
O5	0.0189 (9)	0.0234 (8)	0.0205 (7)	−0.0015 (6)	0.0083 (6)	−0.0010 (6)
O6	0.0403 (11)	0.0190 (9)	0.0315 (8)	0.0094 (8)	0.0124 (8)	0.0088 (7)
O7	0.0239 (9)	0.0271 (8)	0.0131 (7)	−0.0021 (7)	0.0032 (6)	−0.0037 (6)
C1	0.0101 (11)	0.0124 (10)	0.0141 (9)	0.0013 (8)	0.0024 (8)	−0.0006 (7)
C2	0.0142 (11)	0.0138 (10)	0.0128 (9)	−0.0026 (8)	0.0019 (8)	−0.0025 (7)
C3	0.0157 (11)	0.0109 (10)	0.0127 (9)	−0.0035 (8)	0.0062 (8)	−0.0036 (7)
C4	0.0108 (11)	0.0120 (10)	0.0131 (9)	−0.0037 (8)	0.0023 (8)	−0.0016 (7)
C5	0.0157 (11)	0.0134 (10)	0.0139 (9)	0.0007 (8)	0.0060 (8)	0.0007 (7)
C6	0.0163 (12)	0.0156 (10)	0.0144 (9)	−0.0014 (8)	0.0060 (8)	−0.0001 (7)
C7	0.0204 (12)	0.0198 (11)	0.0155 (9)	0.0033 (9)	0.0081 (8)	0.0036 (8)
C8	0.0229 (13)	0.0160 (11)	0.0246 (11)	−0.0028 (9)	0.0115 (9)	0.0002 (8)
C9	0.0233 (13)	0.0142 (11)	0.0171 (10)	0.0022 (9)	0.0098 (9)	−0.0005 (8)
C10	0.0265 (14)	0.0272 (12)	0.0244 (11)	0.0099 (10)	0.0075 (10)	−0.0038 (9)
C11	0.0189 (12)	0.0124 (10)	0.0129 (9)	0.0026 (8)	0.0030 (8)	−0.0010 (7)
C12	0.0244 (13)	0.0212 (11)	0.0137 (9)	−0.0005 (9)	0.0076 (9)	−0.0044 (8)
C13	0.0207 (12)	0.0128 (10)	0.0102 (9)	0.0001 (8)	0.0039 (8)	−0.0013 (7)
C14	0.0203 (12)	0.0221 (12)	0.0150 (9)	−0.0026 (9)	0.0081 (8)	−0.0033 (8)
C15	0.0154 (12)	0.0136 (10)	0.0200 (10)	−0.0006 (8)	0.0081 (9)	0.0016 (8)

### Geometric parameters (Å, °)

**Table:**

Fe1—C15	1.7907 (19)	C4—C5	1.533 (2)
Fe1—C14	1.793 (2)	C4—H4	0.9500
Fe1—C13	1.807 (2)	C5—C7	1.533 (3)
Fe1—C3	2.0410 (18)	C5—C8	1.544 (3)
Fe1—C2	2.0632 (19)	C5—C6	1.547 (3)
Fe1—C1	2.086 (2)	C6—H6A	0.9900
Fe1—C4	2.1171 (18)	C6—H6B	0.9900
O1—C9	1.363 (2)	C7—H7A	0.9800
O1—C1	1.423 (2)	C7—H7B	0.9800
O2—C9	1.197 (2)	C7—H7C	0.9800
O3—C11	1.370 (2)	C8—H8A	0.9800
O3—C3	1.413 (2)	C8—H8B	0.9800
O4—C11	1.195 (2)	C8—H8C	0.9800
O5—C13	1.140 (2)	C9—C10	1.490 (3)
O6—C14	1.144 (2)	C10—H10A	0.9800
O7—C15	1.147 (2)	C10—H10B	0.9800
C1—C2	1.429 (2)	C10—H10C	0.9800
C1—C6	1.517 (2)	C11—C12	1.501 (2)
C2—C3	1.391 (3)	C12—H12A	0.9800
C2—H2	0.9500	C12—H12B	0.9800
C3—C4	1.416 (2)	C12—H12C	0.9800
			
C15—Fe1—C14	100.09 (9)	Fe1—C4—H4	90.6
C15—Fe1—C13	98.05 (9)	C4—C5—C7	110.43 (16)
C14—Fe1—C13	92.40 (9)	C4—C5—C8	107.07 (14)
C15—Fe1—C3	136.08 (8)	C7—C5—C8	108.42 (15)
C14—Fe1—C3	120.64 (8)	C4—C5—C6	109.38 (15)
C13—Fe1—C3	96.05 (8)	C7—C5—C6	110.93 (14)
C15—Fe1—C2	133.11 (8)	C8—C5—C6	110.52 (17)
C14—Fe1—C2	91.64 (8)	C1—C6—C5	110.10 (15)
C13—Fe1—C2	126.85 (8)	C1—C6—H6A	109.6
C3—Fe1—C2	39.62 (7)	C5—C6—H6A	109.6
C15—Fe1—C1	93.24 (8)	C1—C6—H6B	109.6
C14—Fe1—C1	94.73 (8)	C5—C6—H6B	109.6
C13—Fe1—C1	165.38 (8)	H6A—C6—H6B	108.2
C3—Fe1—C1	69.34 (7)	C5—C7—H7A	109.5
C2—Fe1—C1	40.28 (7)	C5—C7—H7B	109.5
C15—Fe1—C4	97.89 (8)	H7A—C7—H7B	109.5
C14—Fe1—C4	160.13 (8)	C5—C7—H7C	109.5
C13—Fe1—C4	93.41 (8)	H7A—C7—H7C	109.5
C3—Fe1—C4	39.77 (7)	H7B—C7—H7C	109.5
C2—Fe1—C4	69.75 (7)	C5—C8—H8A	109.5
C1—Fe1—C4	75.79 (7)	C5—C8—H8B	109.5
C9—O1—C1	120.04 (15)	H8A—C8—H8B	109.5
C11—O3—C3	115.66 (15)	C5—C8—H8C	109.5
O1—C1—C2	110.73 (15)	H8A—C8—H8C	109.5
O1—C1—C6	115.27 (15)	H8B—C8—H8C	109.5
C2—C1—C6	121.04 (16)	O2—C9—O1	123.76 (18)
O1—C1—Fe1	122.01 (12)	O2—C9—C10	126.38 (17)
C2—C1—Fe1	69.01 (11)	O1—C9—C10	109.86 (17)
C6—C1—Fe1	111.37 (13)	C9—C10—H10A	109.5
C3—C2—C1	112.72 (16)	C9—C10—H10B	109.5
C3—C2—Fe1	69.33 (11)	H10A—C10—H10B	109.5
C1—C2—Fe1	70.71 (11)	C9—C10—H10C	109.5
C3—C2—H2	123.6	H10A—C10—H10C	109.5
C1—C2—H2	123.6	H10B—C10—H10C	109.5
Fe1—C2—H2	128.1	O4—C11—O3	123.65 (17)
C2—C3—O3	121.22 (16)	O4—C11—C12	126.61 (18)
C2—C3—C4	116.78 (16)	O3—C11—C12	109.74 (17)
O3—C3—C4	121.78 (17)	C11—C12—H12A	109.5
C2—C3—Fe1	71.05 (11)	C11—C12—H12B	109.5
O3—C3—Fe1	121.47 (12)	H12A—C12—H12B	109.5
C4—C3—Fe1	73.01 (10)	C11—C12—H12C	109.5
C3—C4—C5	117.86 (17)	H12A—C12—H12C	109.5
C3—C4—Fe1	67.22 (10)	H12B—C12—H12C	109.5
C5—C4—Fe1	112.09 (12)	O5—C13—Fe1	176.88 (16)
C3—C4—H4	121.1	O6—C14—Fe1	178.7 (2)
C5—C4—H4	121.1	O7—C15—Fe1	178.33 (18)
			
C9—O1—C1—C2	153.86 (16)	C14—Fe1—C3—O3	−67.18 (18)
C9—O1—C1—C6	−64.1 (2)	C13—Fe1—C3—O3	29.24 (16)
C9—O1—C1—Fe1	76.28 (18)	C2—Fe1—C3—O3	−115.62 (19)
C15—Fe1—C1—C2	172.64 (12)	C1—Fe1—C3—O3	−150.23 (16)
C14—Fe1—C1—C2	−86.95 (12)	C4—Fe1—C3—O3	117.3 (2)
C13—Fe1—C1—C2	32.0 (3)	C15—Fe1—C3—C4	20.20 (17)
C3—Fe1—C1—C2	34.07 (11)	C14—Fe1—C3—C4	175.53 (12)
C4—Fe1—C1—C2	75.32 (11)	C13—Fe1—C3—C4	−88.06 (12)
C15—Fe1—C1—C6	56.35 (13)	C2—Fe1—C3—C4	127.08 (16)
C14—Fe1—C1—C6	156.76 (13)	C1—Fe1—C3—C4	92.47 (12)
C13—Fe1—C1—C6	−84.3 (3)	C2—C3—C4—C5	−46.2 (2)
C3—Fe1—C1—C6	−82.22 (13)	O3—C3—C4—C5	139.23 (17)
C2—Fe1—C1—C6	−116.29 (17)	Fe1—C3—C4—C5	−103.85 (15)
C4—Fe1—C1—C6	−40.97 (12)	C2—C3—C4—Fe1	57.69 (15)
O1—C1—C2—C3	−174.00 (16)	O3—C3—C4—Fe1	−116.92 (17)
C6—C1—C2—C3	46.5 (3)	C15—Fe1—C4—C3	−166.01 (12)
Fe1—C1—C2—C3	−56.50 (14)	C14—Fe1—C4—C3	−11.4 (3)
O1—C1—C2—Fe1	−117.50 (14)	C13—Fe1—C4—C3	95.37 (12)
C6—C1—C2—Fe1	102.98 (17)	C2—Fe1—C4—C3	−32.83 (11)
C15—Fe1—C2—C3	114.62 (13)	C1—Fe1—C4—C3	−74.64 (11)
C14—Fe1—C2—C3	−139.90 (11)	C15—Fe1—C4—C5	−53.89 (14)
C13—Fe1—C2—C3	−45.67 (14)	C14—Fe1—C4—C5	100.7 (2)
C1—Fe1—C2—C3	124.71 (15)	C13—Fe1—C4—C5	−152.51 (13)
C4—Fe1—C2—C3	32.95 (10)	C3—Fe1—C4—C5	112.12 (18)
C15—Fe1—C2—C1	−10.09 (16)	C2—Fe1—C4—C5	79.29 (13)
C14—Fe1—C2—C1	95.39 (11)	C1—Fe1—C4—C5	37.48 (13)
C13—Fe1—C2—C1	−170.38 (11)	C3—C4—C5—C7	170.42 (16)
C3—Fe1—C2—C1	−124.71 (15)	Fe1—C4—C5—C7	95.38 (15)
C4—Fe1—C2—C1	−91.76 (11)	C3—C4—C5—C8	−71.7 (2)
C1—C2—C3—O3	173.19 (16)	Fe1—C4—C5—C8	−146.77 (13)
Fe1—C2—C3—O3	115.93 (16)	C3—C4—C5—C6	48.1 (2)
C1—C2—C3—C4	−1.4 (2)	Fe1—C4—C5—C6	−26.98 (18)
Fe1—C2—C3—C4	−58.71 (15)	O1—C1—C6—C5	−178.32 (15)
C1—C2—C3—Fe1	57.27 (14)	C2—C1—C6—C5	−40.5 (2)
C11—O3—C3—C2	64.8 (2)	Fe1—C1—C6—C5	37.17 (18)
C11—O3—C3—C4	−120.82 (19)	C4—C5—C6—C1	−6.0 (2)
C11—O3—C3—Fe1	150.55 (14)	C7—C5—C6—C1	−128.02 (16)
C15—Fe1—C3—C2	−106.89 (14)	C8—C5—C6—C1	111.68 (17)
C14—Fe1—C3—C2	48.44 (14)	C1—O1—C9—O2	−4.1 (3)
C13—Fe1—C3—C2	144.86 (11)	C1—O1—C9—C10	176.09 (16)
C1—Fe1—C3—C2	−34.61 (10)	C3—O3—C11—O4	4.5 (3)
C4—Fe1—C3—C2	−127.08 (16)	C3—O3—C11—C12	−175.53 (15)
C15—Fe1—C3—O3	137.49 (15)		
